# MEG Biomarkers in relation to plasma neuropathology biomarkers of Alzheimer’s disease

**DOI:** 10.1002/alz.089367

**Published:** 2025-01-09

**Authors:** Alejandra García‐Colomo

**Affiliations:** ^1^ Universidad Complutense de Madrid, Madrid, Madrid Spain

## Abstract

**Background:**

The preclinical stage of Alzheimer’s disease (AD) has gained attention for the window of opportunity it opens for early detection and intervention. Given the high invasiveness of PET and CSF markers, electrophysiology and plasma biomarkers are being studied as alternate biomarkers for early detection and disease tracking. The aim of this study is twofold. First, to address the relationship between a compound centrality score (CS; i.e., a measure of the relevance of a region within the network) and plasma pathology markers of AD in unimpaired individuals with elevated neurofilament‐light chain (NfL) and phosphorylated tau 231 (p‐tau231). Lastly, to evaluate whether this association is greater in hubs.

**Method:**

33 individuals with available MEG recordings and over‐the‐median plasma NfL and p‐tau231 levels were included. A compound CS for each 1210 brain sources of every subject was calculated combining classical centrality metrics. The association between each node’s CS and plasma biomarkers was studied using a correlation analysis. A subsequent correlation between the obtained rho and the grand‐average of the CS was performed to test whether greater associations were found in hubs.

**Result:**

Increasing p‐tau231 concentrations were associated with greater relevance of posterior areas through their theta oscillations and lower relevance of left areas through their gamma oscillations. The most relevant areas are also the ones that present the greatest association between their CS and p‐tau231.

**Conclusion:**

The results of this pioneering study are consistent with previous literature and demonstrate early alterations in network communication associated with elevated plasma biomarkers in cognitively unimpaired individuals, particularly in vulnerable areas to AD pathology.

